# Centenarian clocks: epigenetic clocks for validating claims of exceptional longevity

**DOI:** 10.1007/s11357-023-00731-7

**Published:** 2023-03-25

**Authors:** Eric Dec, James Clement, Kaiyang Cheng, George M. Church, Michael B. Fossel, David H. Rehkopf, Luis Rosero-Bixby, Michael S. Kobor, David TS. Lin, Ake T. Lu, Zhe Fei, Wei Guo, Yap Ching Chew, Xiaojing Yang, Sulistyo E. Dwi Putra, Alex P. Reiner, Adolfo Correa, Adrian Vilalta, Chiara Pirazzini, Giuseppe Passarino, Daniela Monti, Beatrice Arosio, Paolo Garagnani, Claudio Franceschi, Steve Horvath

**Affiliations:** 1 Department of Pediatrics, Division of Genetics and Genomic Medicine, University of California, Irvine, USA; 2 Betterhumans Inc., Gainesville, FL USA; 3 Department of Chemical, Pharmaceutical and Agricultural Sciences, University of Ferrara, Ferrara, Italy; 4 Medical Informatics, David Geffen School of Medicine, University of California Los Angeles, Los Angeles, CA USA; 5 Department of Genetics, Harvard Medical School, Boston, MA USA; 6 Wyss Institute for Biologically Inspired Engineering, Harvard University, Cambridge, MA USA; 7 Telocyte LLC, Grand Rapids, USA; 8 Epidemiology & Population Health and Medicine, School of Medicine, Stanford University, Stanford, CA USA; 9 Centro Centroamericano de Población, Universidad de Costa Rica, San Pedro, Costa Rica; 10 Edwin S.H. Leong Healthy Aging Program, Centre for Molecular Medicine and Therapeutics, University of British Columbia, Vancouver, BC Canada; 11 Dept. of Human Genetics, David Geffen School of Medicine, University of California Los Angeles, Los Angeles, CA USA; 12 Altos Labs, San Diego, CA USA; 13 Dept. of Biostatistics, Fielding School of Public Health, University of California Los Angeles, Los Angeles, CA USA; 14 Zymo Research Corp, Irvine, CA USA; 15 Department of Biology, Faculty of Biotechnology, University of Surabaya, Surabaya, 60293 Indonesia; 16 Public Health Sciences Division, Fred Hutchinson Cancer Research Center, Seattle, WA 98109 USA; 17 Medicine and Population Health Science, University of Mississippi Medical Center, Jackson, USA; 18 Outcomes Metrics LLC, San Diego, CA USA; 19 IRCCS Istituto Delle Scienze Neurologiche Di Bologna, Bologna, Italy; 20 Department of Biology Ecology and Earth Science, University of Calabria, Rende, Italy; 21 Department of Experimental and Clinical, Biomedical Sciences “Mario Serio” University of Florence, Florence, Italy; 22 Department of Clinical Sciences and Community Health, University of Milan, Milan, Italy; 23 Department of Medical and Surgical Sciences, University of Bologna, 40126 Bologna, Italy; 24 Alma Mater Research Institute On Global Challenges and Climate Change (Alma Climate), University of Bologna, Bologna, Italy; 25 Department of Laboratory Medicine, Clinical Chemistry, Karolinska Institutet, Karolinska University Hospital, Stockholm, Sweden; 26 CNR Institute of Molecular Genetics “Luigi Luca Cavalli-Sforza”, Unit of Bologna, Bologna, Italy; 27 Institute of Information Technologies, Mathematics and Mechanics, Lobachevsky State University, Nizhny Novgorod, Russia

**Keywords:** Centenarian clocks, Epigenetic clocks, Longevity

## Abstract

Claims surrounding exceptional longevity are sometimes disputed or dismissed for lack of credible evidence. Here, we present three DNA methylation-based age estimators (epigenetic clocks) for verifying age claims of centenarians. The three centenarian clocks were developed based on *n* = 7039 blood and saliva samples from individuals older than 40, including *n* = 184 samples from centenarians, 122 samples from semi-supercentenarians (aged 105 +), and 25 samples from supercentenarians (aged 110 +). The oldest individual was 115 years old. Our most accurate centenarian clock resulted from applying a neural network model to a training set composed of individuals older than 40. An epigenome-wide association study of age in different age groups revealed that age effects in young individuals (age < 40) are correlated (*r* = 0.55) with age effects in old individuals (age > 90). We present a chromatin state analysis of age effects in centenarians. The centenarian clocks are expected to be useful for validating claims surrounding exceptional old age.

**Supplementary Information:**

The online version contains supplementary material available at 10.1007/s11357-023-00731-7.

## Introduction

In 2016, controversy surrounding the limits of human lifespan ignited when Vijg and colleagues published an analysis of demographic data suggesting a “natural limit” to human life [1]. Shortly thereafter, Brown et al. (2017) [2] challenged Vijg’s assertion after re-analysis of the same data utilizing different statistical techniques and assumptions. Highlights of the history of this debate are well-summarized by Eisenstein’s article published earlier last year [3].

Clouding this debate is poor record keeping in the early twentieth century, and extreme age claims made for secondary gain. Norris McWhirter of the Guinness Book of World Records, wrote, “No single subject is more obscured by vanity, deceit, falsehood, and deliberate fraud than the extremes of human longevity” [4, 5]. Mistakes in age claims can also arise due to dementia or confabulations. The maximum life span of humans is currently determined by Jeanne Calment, documented to have lived for 122 years. Controversy still exists over Jeanne Calment’s age despite verified documentation [6, 7]. Although a sample of her blood is stored and analyses might help resolve the controversy, to date, it has not been allowed due to ethical constraints surrounding the informed consent signed at the time of sample donation [8].

By necessity, many demographic studies of longevity relied on populations for which birth records had been kept. Formal recording of births and deaths in the USA began in Virginia in 1632. But not until 1933, were all states of the USA registering and reporting births and deaths with acceptable event coverage to the bureau of national birth and death statistics [9]. Demographic methodologies and early census activities from 1632 to 1933 were evolving and far from perfect. If a person were born before 1933, it may prove difficult for their age claim to be validated with a US government-issued document. Had Jeanne Calment (born 1875) been born in the USA, her birth might not have been recorded properly, and her case subsequently discarded by demographers. This raises the question, might other supercentenarians be excluded from demographic databases because their age is not verifiable by one or more documents trusted as credible by a demographer? Even with proper documentation, Jeanne Calment’s age is doubted because documentation alone can be falsified or misattributed to an heir as some have suggested [7]. In theory, a very accurate molecular estimator of age could settle such disputes and even obviate the need for birth certificates thereby allowing more inclusive sample collections from countries with incomplete census systems.

Highly accurate age estimators can be built based on DNAm levels [10–15]. The high accuracy of epigenetic clocks has been replicated numerous times and would be one way to verify the age of individuals too old to have been counted accurately by nascent census methods [16–18]. However, most current epigenetic clocks underestimate the ages of older individuals (due to the well-known regression to the mean effect) and lead to relatively low age correlations in the oldest old [19–21]. Here, we present three new epigenetic clocks whose express purpose is to verify age claims from centenarians.

## Results

### Data

We combined DNAm data from multiple sources. Centenarians and their offspring are collected by James Clement. Centenarian samples were obtained from multiple sources including from Italy [19] as well as blood samples from several large cohort studies including the Framingham Heart Study and Women’s Health Initiative (Table 1). The clocks were trained based on *n* = 7039 blood and saliva samples from individuals older than 40, including *n* = 184 individuals older than 100, 122 individuals older than 105, and 25 individuals older than 110. The oldest individual in the training data was over 115 years of age (Table 1).

**Table 1:** Characteristics of study individuals in training and test data sets

				Tissue			
Age group	*N*	Female	Age	Blood	Saliva	Buccal	Urine
Training data 1							
Age ≥ 110	25	20 (80.0%)	111.9 ± 1.4 [110, 115]	20 (80.0%)	2 (8.0%)	3 (12.0%)	0 (0%)
Age ≥ 100	184	128 (69.6%)	105.6 ± 3.5 [100, 115]	175 (95.1%)	6 (3.3%)	3 (1.6%)	0 (0%)
Age ≥ 90	358	220 (61.5%)	100.0 ± 6.6 [90, 115]	349 (97.5%)	6 (1.7%)	3 (0.8%)	0 (0%)
Age ≥ 80	1262	672 (53.2%)	88.2 ± 8.5 [80, 115]	1253 (99.3%)	6 (0.5%)	3 (0.2%)	0 (0%)
Age ≥ 40	7039	3838 (54.5%)	68.9 ± 13.1 [40, 115]	7030 (99.9%)	6 (0.1%)	3 (0.0%)	0 (0%)
Training data 2							
Age ≥ 100	184	128 (69.6%)	105.6 ± 3.5 [100, 115]	175 (95.1%)	6 (3.3%)	3 (1.6%)	0 (0%)
Urine Test data							
Age [50, 84]	12	7 (58.3%)	65.6 ± 10.7 [52, 84]	0 (0%)	0 (0%)	0 (0%)	12 (100%)
Age [2, 49]	22	10 (45.5%)	28.7 ± 13.2 [2, 48]	0 (0%)	0 (0%)	0 (0%)	22 (100%)

The table summarizes the characteristics of individuals in training data 1 (*n* = 8868), training data 2 (*n* = 184), and test data (*n* = 34). DNA methylation data were profiled in multiple tissue types in the training data set and profiled in urine in the test data set. Age is presented in the format of mean ± SD [range]. Female and tissue type variables are presented in the format of count (percentage %)

To reduce the computational burden of our neural network predictors, we applied strong pre-filtering steps resulting in 33,495 individual cytosines (Methods). Our subsequent analyses focused on the same set of 33,495 CpGs.

### Standard epigenetic clocks underestimate ages of centenarians

We applied 3 widely used epigenetic age estimators to the data set (Supplementary Fig. S1).

The blood based clock by Hannum [12], the pan tissue clock (Horvath 2013) [11], and the skin and blood clock [15]. When looking at the entire age range (from 40 to 115), we observe high Pearson correlations between DNAmAge and age (*r* = 0.89, *r* = 0.88, *r* = 0.92, Supplementary Fig. S1A, D, G). Similarly, we observe high age correlations in individuals aged 80 or more (*r* = 0.69, *r* = 0.73, *r* = 0.80, Supplementary Fig. S1C, F, I). However, these clocks greatly underestimate the ages of centenarians (median errors 18 years, 12 years, and 15 years, Supplementary Fig. S1B, E, F).

The regression to the mean effect (which incidentally gives regression analysis its name) explains why the epigenetic clocks tend to underestimate ages of individuals that are very old.

Our centenarian clocks greatly outperform these standard clocks as described in the following.

### Centenarian clocks

DNA methylation clocks are defined as prediction methods that regress a transformed version of chronological age (outcome variable) on methylation levels at cytosine-phosphate-guanines (CpGs). We developed three different clocks for centenarians that differ along two dimensions (i) the age range in the training sets and (ii) the method of machine learning: elastic net (EN) regression or neural network (NN).

**Fig. 1:**
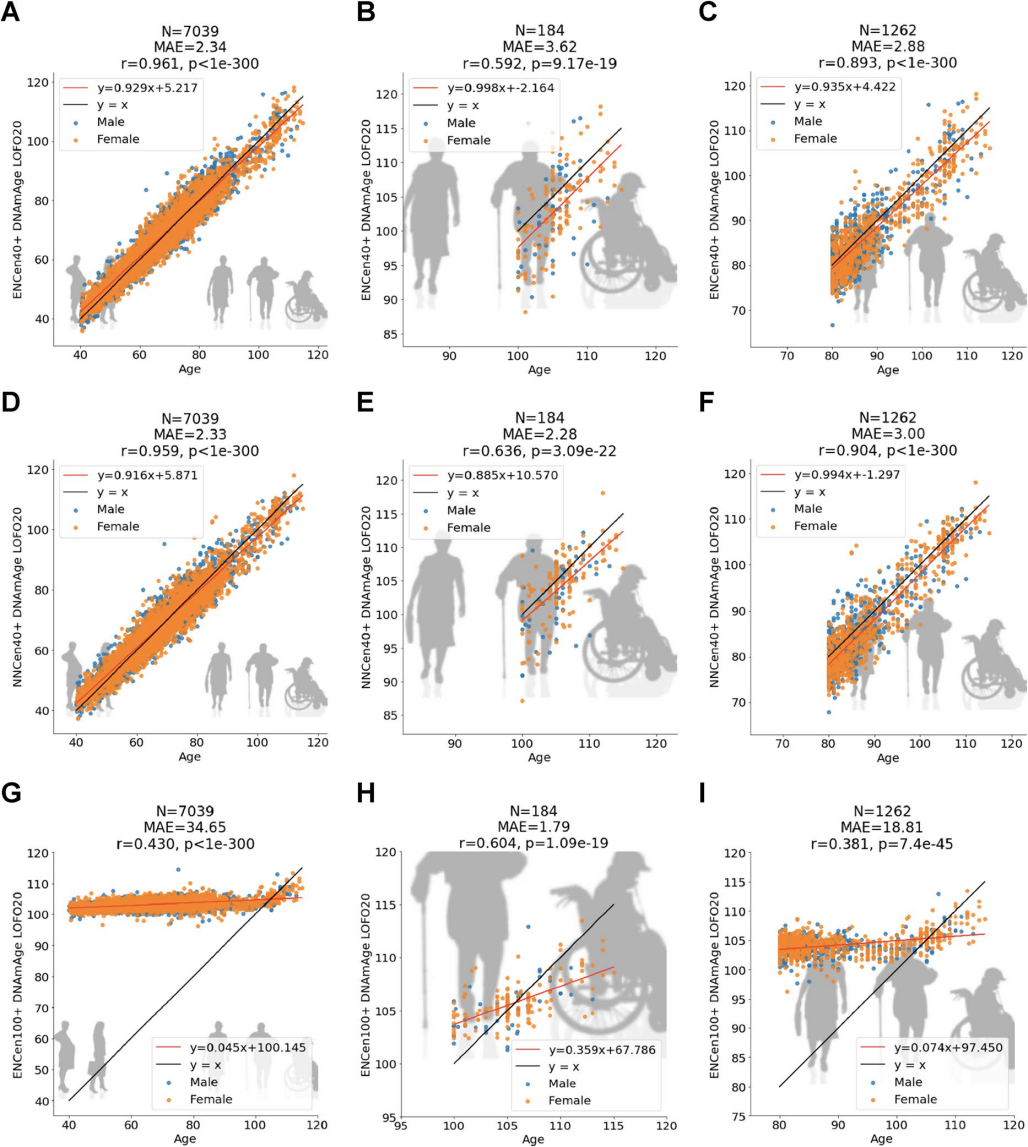
Cross-validation analysis of three epigenetic clocks for centenarians. Age estimation 20-fold cross-validation (LOFO20) of the ENCen40 + , ENCen100 + , and NNCen40 + clocks in blood, saliva, and buccals cells, for different age ranges (columns). The panels relate chronological age (*x*-axis) to DNAm age estimates (*y*-axis) from the ENCen40 + (**A**, **B**, **C**) and NNCen40 + (**D**, **E**, **F**), and ENCen100 + (**G**, **H**, **I**), respectively. Each column corresponds to a different age range. DNA methylation data from age 40 to 115 (**A**, **D**, **G**), 100 to 115 (**B**, **E**, **H**), and 80 to 115 (**C**, **F**, **I**). Each panel reports the sample size (N), the median absolute error (MAE), Pearson correlation coefficient (*r*), the *p* value (*p*), and each point is color coded by sex (blue = male)

We explored several different age ranges when it comes to developing centenarian clocks. For the sake of brevity, we report results for two scenarios: individuals aged 40 years or older (denoted 40 +) and individuals aged 100 years or older (denoted 100 +). Despite the inclusion of middle aged individuals, the resulting predictors deserve the label “Centenarian clock” due to their relatively high cross-validation estimates of the Pearson correlation between chronological age and its DNAm-based estimate (*R* ≥ 0.59) in centenarians (Fig. 1B, E, H). The Pearson correlation is high given that the underlying age range (from 100 to 115 years) is very narrow.

The centenarian clocks lead to high age correlations in the 80 + group where we observe *R* = 0.89 for the ENCen40 + clock and *R* = 0.90 for the NNCen40 + clocks (Fig. 1C, F).

We managed to fit an elastic regression model in centenarians (100 + age group), which led to the elastic net clock denoted as ENCen100 + . However, a neural network-based clock could not be developed in this 100 + age group since the model failed to converge in this small data set.

As expected, the elastic net (ENCen40 +) and neural net (NNCen40 +) clocks trained in individuals 40 + work substantially better in non-centenarians compared to the ENCen100 + clock (Fig. 1A, D, G). The neural network based clock (NNCen40 +) achieved higher accuracy than the elastic net based clock (ENCen40 +) in terms of correlation and absolute error (*r* = 0.636 and MAE = 2.28 years for NNCen40 + clock compared to *r* = 0.592 and MAE = 4.31 for ENCen40 + , Fig. 1B, E).

For most situations, we advise against the use of the ENCen100 + clock, but it may be useful for evaluating supercentenarians (i.e., individuals older than 110). ENCen100 + , which was trained only on centenarian samples, leads to a lower median absolute error in centenarians than the other clocks (MAE = 1.8 years for ENCen100 + compared to MAE = 2.28 for the NNCen40 + clock, Fig. 1E, H), but it is inferior in terms of the age correlations (*r* = 0.604, ENCen100 + compared to *r* = 0.636 for the NNCen40 + clock, Fig. 1E, H).

By comparing Fig. 1 with Supplementary Fig. S1, one can see that the centenarian clocks are better calibrated than the original clocks. Centenarians still appear to be epigenetically younger than their chronological age according to the cross-validation-based estimates of DNAm age (Fig. 1). This underestimate is expected based on the well-known regression-to-the-mean effect.

### Urine samples

**Fig. 2:**
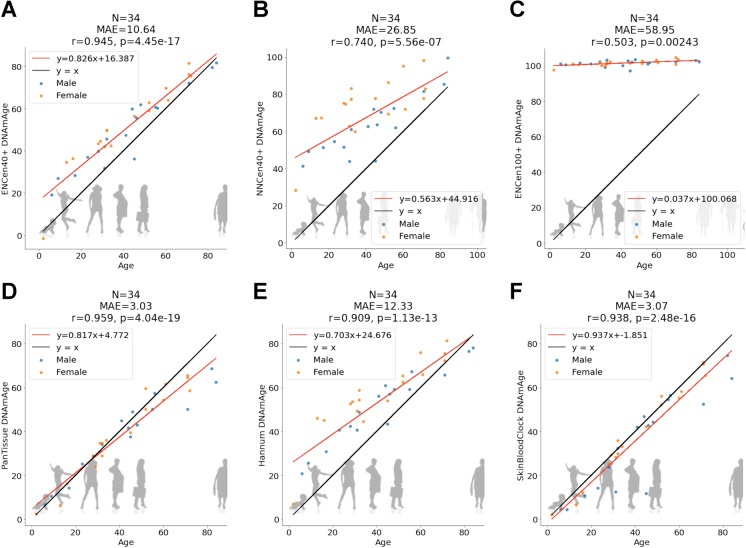
Urine samples. Age estimation of the ENCen40 + , ENCen100 + , and NNCen40 + clocks. The panels relate chronological age (*x*-axis) to DNAm Age estimates (*y*-axis) from the ENCen40 + (**A**) and ENCen100 + (**B**), and NNCen40 + (**C**), respectively. Results for the (**D**) pan tissue clock (Horvath 2013), (**E**) Hannum blood clock (Hannum), and (**F**) skin and blood clock [15]

Due to their frailty, it is sometimes difficult to collect blood from older people. By contrast, the collection of urine is arguably less invasive. Therefore, we explored whether our epigenetic clocks also apply to urine samples. We find that ENCen40 + clock leads to the highest age correlation in urine (*r* = 0.945, Fig. 2), followed by the neural network-based clock NNCen40 + (*r* = 0.74). Since the urine samples were collected from people aged less than 90, we also find that previously published clocks are accurate as well, e.g., the pan tissue clock (*r* = 0.96), Hannum blood based clock (*r* = 0.91), and the skin and blood clock (*r* = 0.94, Fig. 2).

### Out of distribution analysis

Our training set involved individuals aged between 40 and 115. This leads to the question how well these clocks generalize to individuals who are younger than 40 (younger than expected) or older than 115 (older than expected). To address this issue, we carried out an “out-of-distribution prediction analysis” using clocks that were trained in different age intervals (Supplementary Fig. S2). In brief, we find that that the clocks are well-aligned/calibrated in younger individuals but lead to relatively strong offset (underestimate) in older individuals (Supplementary Fig. S2). Based on these results, we expect that our centenarian clocks will underestimate the ages of individuals aged above 115.

#### Centenarian clocks weakly predict mortality risk

To assess whether our centenarian clocks are associated with human mortality risk in middle aged individuals, we used methods and data sets from retrospective epidemiological cohort studies as described previously [22–25].

To remove the confounding effect of age, we defined measures of epigenetic age acceleration (AgeAccel) by regressing DNAm age estimate on chronological age and forming raw residuals. The resulting residuals are not correlated with chronological age (*r* = 0). By definition, these residuals are not correlated with chronological [13].

First, we evaluated whether race/ethnicity has a confounding effect. Age acceleration based on the neural network-based clock (NNCen40 +) was not associated with race (Supplementary Fig. S3C), but the other 2 centenarian clocks showed significant (but weak) associations with race (*p* = 0.0025 and *p* = 1.6 × 10^−6^ for Supplementary Fig. S3A, B).

Therefore, we decided to stratify the analysis by race and cohort. Within each stratum defined by race and study cohort, our Cox regression analysis for time to death (due to all-cause mortality) was adjusted for age, sex, and batch effect as needed. The individual Cox regression results were combined via meta-analysis (inverse-variance weighted fixed-effect models). All three centenarian clocks were significantly associated with human mortality risks (Supplementary Fig. S4). The associations are statistically significant for both centenarian clocks trained in the 40 + age group (*P* = 4.8 × 10^−16^ for NNCen40 + and *P* = 9.0 × 10^−8^ for ENCen40 +) and to a lesser extent for the centenarian clock trained in individuals 100 + (*P* = 0.027 for AgeAccelENCen100 + , Supplementary Fig. S4). But the hazard ratios are relatively small: a 1-year increase in epigenetic age acceleration (AgeAccel) is associated with hazard ratios ranging from 1.04 to 1.05 (Supplementary Fig. S4).

#### Centenarian clocks weakly relate to clinical biomarkers

One novel hallmark of epigenetic clocks is their associations with a broad category of age-related conditions and lifestyle factors including diet [22, 26]. Here, we examined the cross-sectional relationship between our clocks and a total of 59 variables including (i) 27 self-reported dietary variables; (ii) 9 biomarkers measuring vegetable consumption dietary (such as carotenoid levels); (iii) 17 clinical biomarkers of organ function, vital signs, metabolic traits, inflammatory markers, cognitive function, lung function, central adiposity, and leukocyte telomere length (LTL); and (iv) 6 lifestyle factors such as education.

We used a robust correlation test (biweight midcorrelation, bicor) that is less sensitive to outliers [27]. We applied the bicor analysis stratified by gender and racial group to 3 large cohorts: FHS, WHI, and JHS, respectively. The results were combined via fixed effect meta-analysis (Methods). Age acceleration for our centenarian clocks trained in individuals 40 + (i.e., ENCen40 + and NNCen40 +) exhibited far more significant associations with these biomarkers compared to the clock trained in individuals 100 + (Supplementary Fig. S5). Both ENCen40 + and NNCen40 + show very similar correlation patterns with the biomarkers (Supplementary Fig. S5). For instance, higher beta-carotene levels are negatively correlated with AgeAccelENCen40 + (bicor =  − 0.17 and *P* = 9.2 × 10^−3^) and AgeAccelNNCen40 + (bicor =  − 0.16 and *P* = 0.017). AgeAccelENCen40 +  is correlated with markers of inflammation, metabolic syndrome, and C-reactive protein (bicor = 0.08 and *P* = 8.8 × 10^−11^) and insulin levels (bicor = 0.09 and *P* = 1.7 × 10^−5^). AgeAcelENCen40 +  increases with glucose, triglyceride, and systolic blood pressure with less extent correlation (bicor ~ 0.04) and decreases with a lung function biomarker indicated by forced expiratory volume in one second (FEV1). We also found that AgeAccelENCen40 + was sensitive in response to body fat distributions such as body mass index (bicor = 0.09 and *P* = 2.2 × 10^−12^) and waist to hip ratio (bicor = 0.08 and *P* = 5.6 × 10^−9^). These results echo our previous findings for the association between epigenetic clocks and body fat distributions [22, 25, 28].

Despite our large sample size, AgeAccelENCen40 + exhibits only nominally significant associations with educational level, income, and handgrip strength (*p* < 0.05, Supplementary Fig. S5). In practice, these 3 variables can be ignored when estimating the ages of centenarians.

### Epigenome-wide association study of age

In our epigenome-wide association study (EWAS), we correlated each individual CpG with chronological age using a Pearson correlation test (implemented in the WGCNA function standardScreeningNumericTrait) [27]. The study was conducted in three age groups: (i) “young” denotes individuals aged between 0 and 40 years (*n* = 344), (ii) “middle” denotes individuals aged between 40 to 90 years (*n* = 6695), and (iii) “old” denotes individuals aged between 90 to 115 (*n* = 252). Our conclusions remain qualitatively the same for different choices of age intervals.

**Fig. 3:**
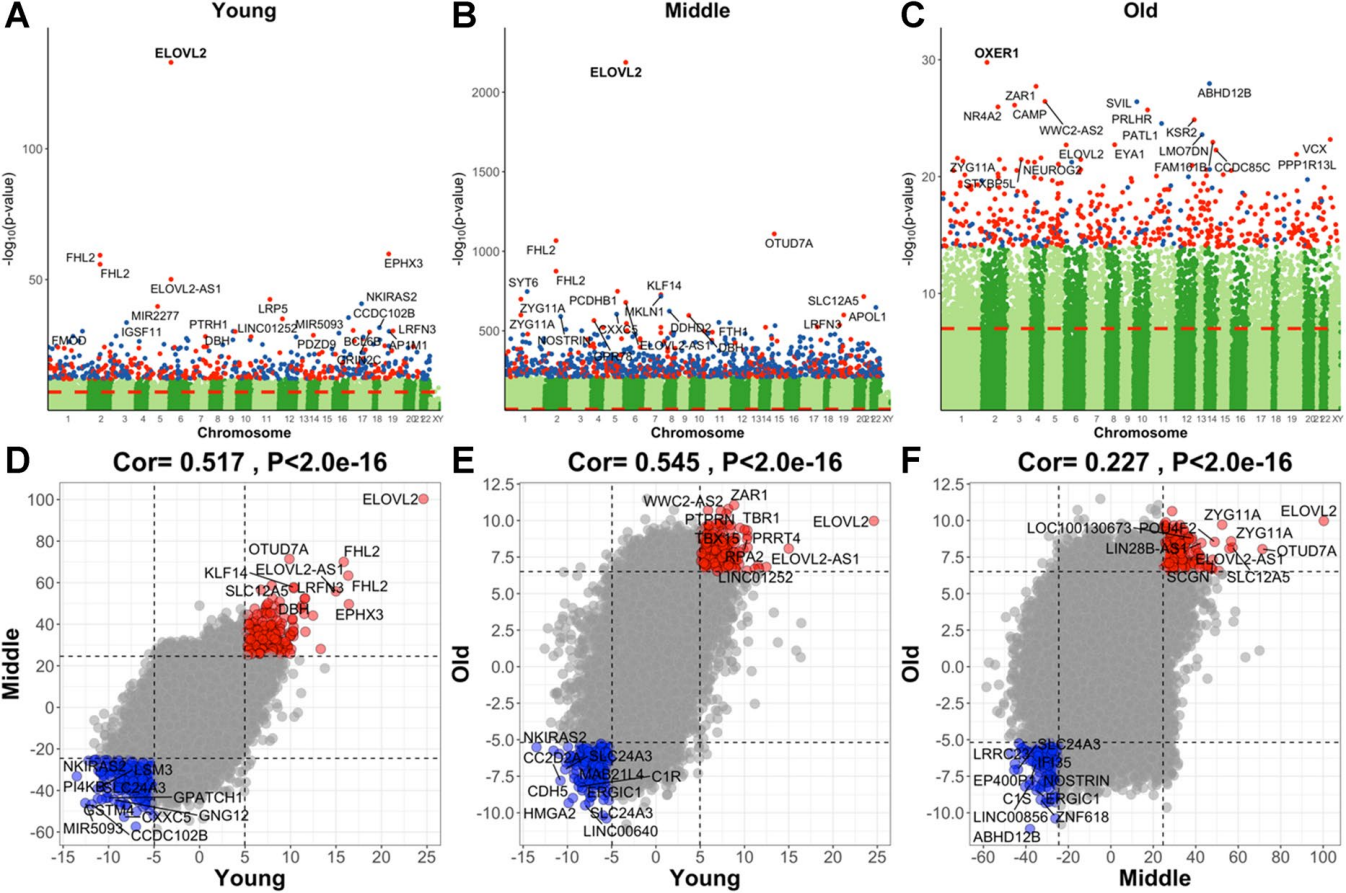
Epigenome-wide association study (EWAS) of age in 3 different age ranges. Epigenome-wide association (EWAS) of age in three age groups: (1) young for age between 0 and 40 (*n* = 344), (2) middle for age between 40 and 90 (*n* = 6695), and (3) old for age between 90 and 115 (*n* = 252), using the training set. **A**–**C** Manhattan plots where the y-axis reports log (base 10 transformed) versions of nominal, unadjusted two-sided *p* values. The red dashed line indicates genome-wide level of significance at *P* < 1.0 × 10^−7^. The *x*-axis displays the coordinates of CpGs in the human Hg19 assembly. The top 1000 CpGs are colored in red and blue if they exhibit highly significant positive and negative age correlations according to *P* < 1.0 × 10^−12^,1.0 × 10^−210^, and 1.0 × 10^−14^ for **A**–**C**, respectively. Adjacent genes are annotated for the top 20 CpGs*.* The gene symbol for the most significant CpG is marked in bold. The lower panels (**D**–**F**) display pairwise correlation among the three EWAS results: **D** young (*x*-axis) versus middle (*y*-axis), **E** young versus old, and **F** middle versus old. Each axis reports a *Z* score. Each dot corresponds to a CpG. Labels are provided for the top 10 hypermethylated/hypomethylated CpGs according to the product of *Z* scores in *x*- and *y*-axis. The Pearson correlation coefficient and corresponding nominal (unadjusted) two-sided correlation test *P* value can be found in the title

Our EWAS was limited to 33,495 CpGs (Methods). Our EWAS results show that chronological age has abundant effects on the methylation levels of our CpGs in different age groups (Fig. 3A-C). The CpG with the highest and most significant correlation with age in the young and middle age group is cg16867657 (*P* = 9.5 × 10^−69^ in the young group and *P* = 5.3 × 10^−2188^ in the middle group). This positively age-related CpG is located on chromosome 5 in *ELOVL2*. A less significant but noteworthy CpG (cg04875128) is located on chromosome 15 near *OTUD7A* (*P* = 5.8 × 10^−1110^).

We find a high conservation of age effects between the young and the old group (*r* = 0.545, Fig. 3E). Surprisingly, the aging effects in middle aged individuals (aged between 40 and 90) were less correlated with those in older individuals (*r* = 0.227, Fig. 3F) which probably reflects limitations of the marginal correlation analysis, which ignored the heterogeneity of the underlying data (data from multiple different cohorts comprised of different groups).

Our EWAS in old individuals (aged between 90 and 115) reveals the following top hits. Noteworthy genes include *OXER1* (oxoeicosanoid receptor 1), which is associated with chemoattraction, inflammation, and oncogenesis. *ZAR1* (zygote arrest protein 1) was first characterized and named according to its role in the oocyte to embryo transition [29]. ZAR1 can inhibit cell cycle progression and may serve as a tumor suppressor [29, 30]. *NR4A2* (nuclear receptor subfamily 4 group A member 2) has been implicated in immune homeostasis via regulatory T cell development [31]. NR4A2 regulates certain aspects of autophagy and some of the nuclear-encoded mitochondrial genes [32]. The *CAMP* (cathelicidin antimicrobial peptide) gene encodes a protein involved in innate immunity against viruses and more generally functions in chemotaxis and inflammatory response regulation. *CAMP* gene promoter methylation inhibits inflammation and induces chondrocyte apoptosis which is known to play a role in osteoarthritis [33]. The CAMP protein has been suggested to be both anti- and protumorigenic depending on the cell type under investigation [34].

### Chromatin state analysis of EWAS results

**Fig. 4:**
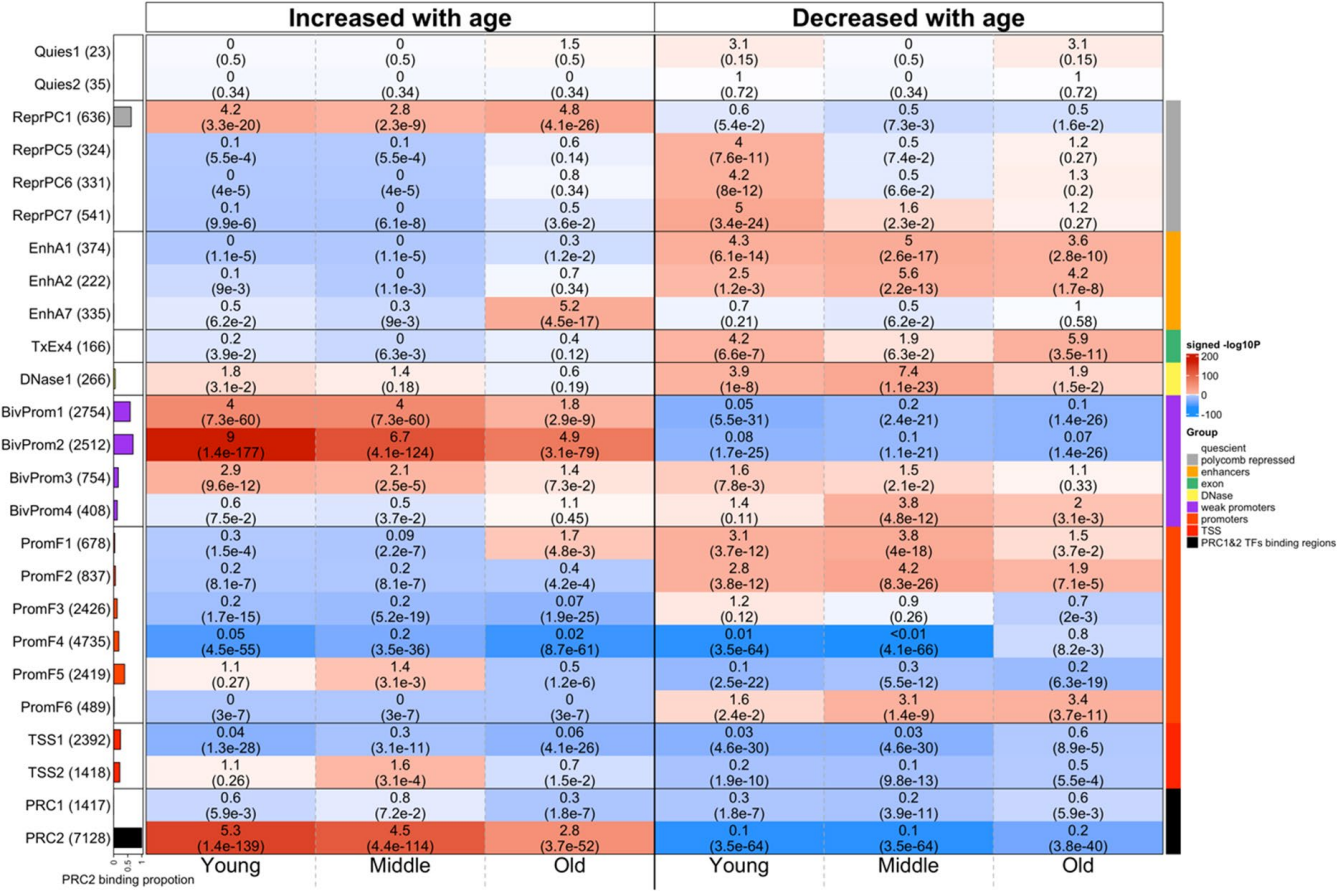
Chromatin state analysis of age-related CpGs. Chromatic state annotation and polycomb repressive complex (PRC) annotation for the age-related CpGs identified from EWAS of age in three age groups: (1) young for age between 0 and 40, (2) middle for age between 40 and 90, and (3) old for age between 90 and 115. The heatmap color codes the hypergeometric overlap analysis between age-related CpGs (columns) and (1) universal chromatin states analysis [33] and PRC1-/PRC2-binding sites defined based on ChipSeq data sets in ENCODE [34]. For each row, the table reports odds ratios (OR) from hypergeometric test results for the top 1000 CpGs that increased/decreased with age from our EWAS of age in three age groups (young, middle, old). The color gradient in the heatmap is based on − log10 (unadjusted hypergeometric *P* value) multiplied by the sign of OR greater than one. Red colors denote OR greater than one in contrast with blue colors for OR less than one. Legend lists states based on their group category. The *y*-axis lists the chromatin state or PRC binding and the respective number of CpGs inside parentheses. The bar plot on the left reports the proportion of CpGs that are known to be bound by PRC2 that ranges from zero (PRC1) to one (PRC2). The left/right panel lists the results based on the top 1000 CpGs with positive and the top 1000 CpGs with negative age correlations, respectively. We displayed 23 universal chromatin states that show significant enrichment/depletion at an uncorrected/nominal *P* < 1.0 × 10^−10^ in any of the EWAS

To characterize the chromatin states in which age-related CpGs are located, we employed a detailed universal chromatin state map constructed based on 1032 experiments that mapped 32 types of chromatin marks in over 100 human cell and tissue types [33] (Fig. 4). We overlaid the positions of the top 1000 positively age-related CpGs and the top 1000 negatively age-related CpGs onto this universal chromatin state map (Supplementary Data 1). We performed a hypergeometric enrichment test with background a background set of 33,340 CpGs that could be mapped to chromatin states (Methods).

In the following, we focus on chromatin states that are enriched with age-related changes in old individuals (age > 90), but most comments apply to EWAS results in younger age groups as well (which reflects the high conservation of age effects across age groups, Fig. 3D-F).

Positive age-related CpGs showed strong enrichments in chromatin states that were previously shown to have a strong association with binding sites of polycomb repressive complex 2 (states BivProm1-2, ReprPC1)(35) [33]. These CpGs localized to PRC2-binding sites are characterized by Eed, Ezh2, and Suz12 binding. PRC2 is a transcriptional repressor complex which is associated with the histone mark H3K27me3 [35]. Significantly, PRC2-mediated methylation of H3K27 is essential for the establishment of bivalent promoters, which simultaneously contain both H3K27me3 and H3K4me3 marks. There is greater enrichment of these CpGs in a bivalent promoter state that contains more H3K27me3 than H3K4me3 (state BivProm2), compared to states such as BivProm1, which contains equal amounts of these two histone types. These results echo those from many human studies that reported age-related gain of DNAm in PRC2-binding sites [36, 37]. We also observe significant enrichment in active enhancer state EnhA7, but this enrichment is somewhat suspect since it could not be observed in our EWAS of middle-aged individuals. The overlap with PRC2-binding sites depends to some extent on age group. The strongest and weakest overlap could be observed in young individuals (age < 40) and old individuals (age > 90), respectively. Target sites of polycomb repressive complex 1 (as opposed to 2) did not overlap significantly with positively age-related CpGs.

Negatively age-related CpGs (those that lose methylation levels with age) led to less significant overlaps with chromatin states. Age-related loss of methylation could be observed for CpGs located in the following chromatin states: first, active enhancer states (EnhA1, EnhA2) associated with H3K4me1, accessible chromatin, H2A.Z, and/or H3K27ac; second, exon-associated transcription state TxEx4 which is most highly enriched for transcription termination sites, exons, and promoters [33]; and third, flanking promoter state PromF6 which was located within 2 kb of annotated transcriptional start sites. The findings are consistent with those from the EWAS of age by the Mammalian Methylation Consortium [38].

### Non-linear relationships between DNAm and age

**Fig. 5:**
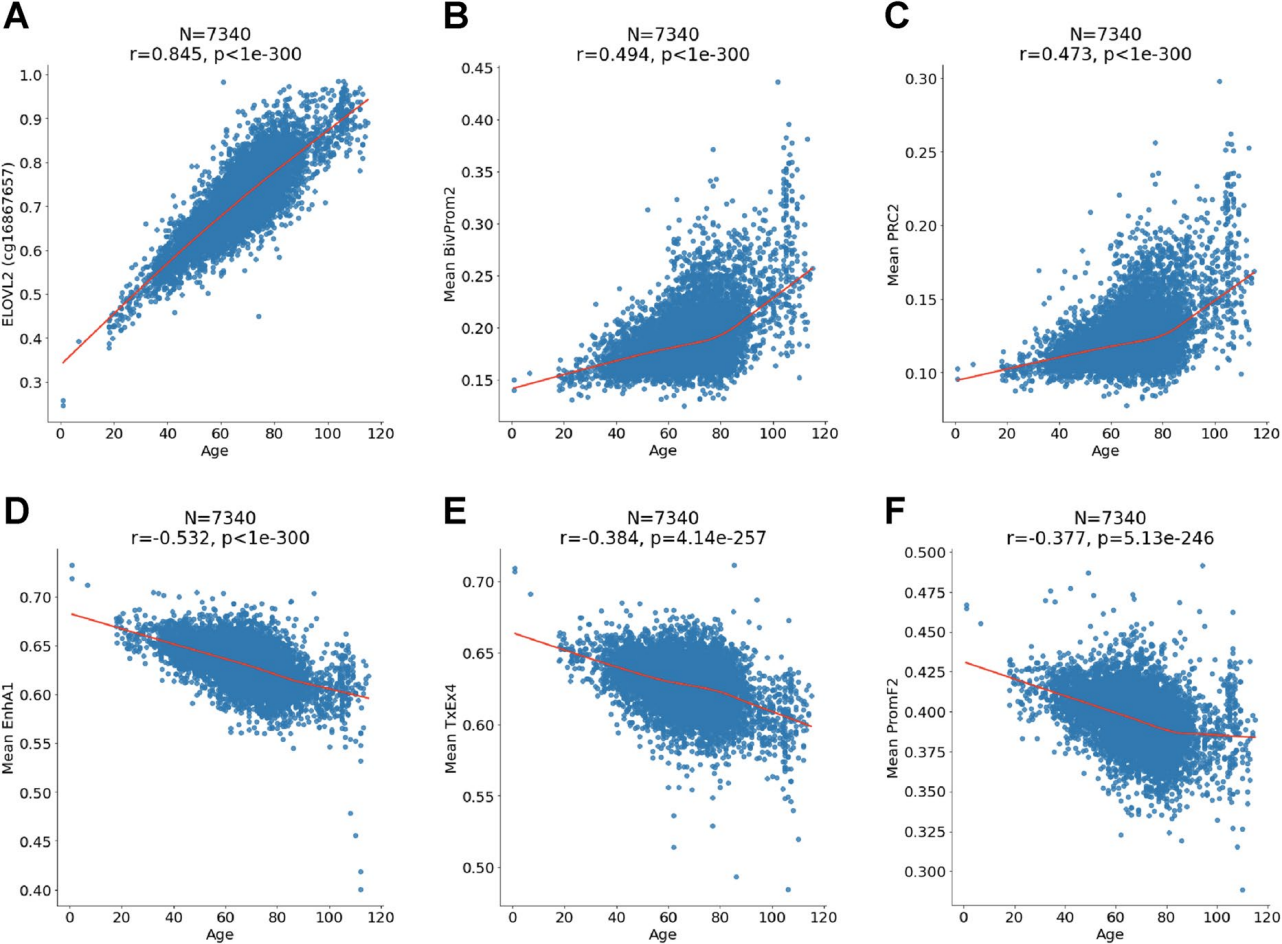
Individual CpGs and mean CpG in chromatin states. Chronological age (*x*-axis) versus **A** ELOVL2 methylation (*y*-axis) or mean methylation in **B** chromatin state BivProm2, **C** target sites of polycomb repressive complex 2, **D** chromatin state EnhA1, **E** chromatin state TxEx4, and **F** chromatin state PromF2. Each panel reports the sample size (*N*), Pearson correlation coefficient (*r*), and the *p* value (*p*), and red line is the LOWESS regression smooth curve

Age can have a non-linear effect on individual CpGs. Similarly, non-linear effects could also be present for the mean methylation levels of CpGs of a given chromatin state. To investigate non-linear patterns, we used LOWESS regression models to the most significant CpGs and chromatin states (Fig. 5). The CpG at *ELOVL2* is strongly linearly correlated with age (Fig. 5A). However, we find evidence for non-linear relationships for the mean methylation levels in chromatin state BivProm2 and binding sites of PRC2 (Fig. 5B,C). Only one chromatin state (PromF2) exhibits a saturation type behavior for age-related loss of DNAm (Fig. 5F). The other chromatin states do not reveal any leveling off effect (Fig. 5).

### Principal component analysis

We carried out a principal component analysis of the DNAm data (Supplementary Fig. S6) to understand the main sources of variation. The first 2 principal components (which explain 19% and 14% of the variation, respectively) reveal two main clusters which correspond to sex (male, female). The first three PCs do not seem to relate to the underlying data source, but principal components 4 and 5 relate weakly to at least one of the underlying data sets (Supplementary Fig. S6B, C). Overall, this analysis shows the expected results: DNA methylation is subject to batch effects. However, age has a far stronger signal as demonstrated by our ability to find CpGs and chromatin states that had previously been found in other data sets.

## Discussion

Centenarians are paragons of successful aging that manage to delay or resist the onset of age-related diseases [39, 40]. Studying these successful agers promises to elucidate the molecular secrets underlying healthy aging. By necessity, previous studies of centenarians were limited to populations that maintained rigorous birth records. In theory, birth certificates could be replaced by accurate molecular age estimates. In practice, it is very challenging to find biomarkers that are accurate and not confounded by biological factors including genetic ancestry, various disease states, or stress factors. As a first step to address this challenge, we propose centenarian clocks that apply to ethnically diverse study participants since they were trained in individuals from different ancestries including European, African, and Hispanic ancestry.

These centenarian clocks were developed as chronological age estimators (i.e., first generation clocks) as opposed to morbidity risk estimators (second generation clocks). In general, epigenetic age estimates can be influenced by conditions such as Down syndrome [41, 42], HIV [43], obesity [44], diet [26], and onset of menopause [45]. Most epigenetic clocks predict human mortality risk when the sample size is sufficiently large [13,22,23,46]. Even our centenarian clocks predict human mortality risk and relate to various clinical biomarkers, but these associations are very weak and have only a negligible effect on the age estimate. This reflects that the centenarian clocks were designed for the purpose of estimating chronological age as opposed to biological age.

Our EWAS of age finds highly significant associations for CpGs located near the following genes. *ELOVL2* (elongation of very long-chain fatty acids-like 2) is an enzyme located in the endoplasmic reticulum membrane. It catalyzes the first and rate limiting step in long-chain fatty acid elongation. CpGs near *ELOVL2* are part of many methylation-based clocks [47]. *ELOVL2* has been reported to play a role in retinal physiology and age-related macular degeneration [48].

Our chromatin state analysis reveals that mean methylation of PRC2 target sites continue to increase late in life while exhibiting increased variability (Fig. 5D). Similarly, the mean methylation levels of two negatively age-related chromatin states (EnhA1, TxEx4) do not exhibit any leveling off effect late in life (Fig. 5). These results suggest that one will be able to build accurate centenarian clocks for people who live beyond 120 years.

Our study has several limitations. First, we removed severe outliers from the training set. This data cleaning step was necessary since we only had a limited number of centenarians. It will be desirable to validate our claims in independent data. Our freely available software can be found in the Supplement. Second, we applied strong pre-filtering steps which limited our analysis to 33,495 individual cytosines. Third, our analysis (both regression and EWAS) ignored batch effects arising from lumping together data from different cohorts. Our principal component analysis reveals evidence for batch effects (Supplementary Fig. S6). Technical confounders are expected to bias the results toward the null hypothesis of zero correlation. In general, this source of confounding is negligible compared to the effect of age on methylation as can be seen from the following two facts. First, our EWAS of age highlights genes (e.g., *ELOVL2*) that have been reported in many previous articles. Second, our chromatin state analysis highlights chromatin states (bivalent promoters, PRC2 bound regions) that have been implicated in previous articles.

Fourth, our enrichment analysis for chromatin states did not adjust for potential biases arising from sequence context (e.g., transcription start site/gene promoter bias or CpG island bias). To address this source of bias, one could use the eForge software tool [49]. Fifth, neural network clocks are vulnerable to overfitting in this relatively small data set as can also be seen in the urine data.

In conclusion, we demonstrate that one can build accurate epigenetic clocks for validating age claims in centenarians. Going forward, we believe that more accurate centenarian clocks can be developed once larger training sets become available.

## Methods

### Ethics

Since the data came from multiple sources, we report multiple ethics approvals. The study was approved through UCLA IRB#18–000315. Betterhumans Inc. received IRB approval (Protocol Number: BH-SC-300, Approval Number: IRCM-2018–185, Approval Date: April 18, 2018). The Italian study was approved by the local ethical committee (S. Orsola Hospital—University of Bologna; Prot. n. 2006061707, amendment 08/11/2011; Fondazione IRCCS Cà Granda Ospedale Maggiore Policlinico, Prot. n. 2035, amendment 30/11/2011; University of Calabria 9/9/2004 amendment on 24/11/2011). A written informed consent form was obtained from all participants. Each of the epidemiological cohort studies was approved by their local ethical committee as detailed in the respective publications describing the data.

### Methylation arrays

We used data generated on two Illumina methylation array platforms: Infinium 450 K array and the Infinium methylation EPIC beadchip array that profiles > 866 k CpGs. The data came from multiple teams that used different normalization methods. To reduce the computational burden, we pre-filtered the CpGs. We focused on CpGs that were part of the following 3 groups: strong positive correlation with age, strong negative correlation, or close to zero correlation with age.

### Removal of outlying samples

Since influential outliers can severely impair the fit of regression models, we erred on the side of caution by removing putative outliers. This was done to minimize the potential effect of platemap errors, human labeling errors, and the presence of blood cancers. To address the concern that the removal of outliers might have led to a biased evaluation, we release the software code in the Supplement. Independent data from centenarians and younger individuals are needed to further evaluate our centenarian clocks.

### Penalized regression models

Penalized regression models (implemented in the R package glmnet [50]) and Python package Sklearn [51] were used to regress chronological age on the CpG probes in the training set. The alpha parameter of glmnet was chosen as 0.5 (elastic net (EN) regression) and the lambda values were chosen using cross-validation on the training data. DNAm age was defined as predicted age. Since samples from centenarians were relatively rare, we used a sample weight of 10 for these samples in our regression models (e.g., weight parameter in glmnet). Our 20-fold cross-validation model splits the data into 20 bins. Circling through the bins, each model was trained in 19 bins and evaluated in the left-out bin. This led to cross-validation-based estimates of the Pearson correlation between age and its methylation-based estimate and the median absolute error.

### Neural network models

Neural network (NN) models (implemented in Python package Sklearn) were used to regress chronological age on the CpG probes in the training set. The hyperparameters (architecture, batch-size, learning rate) of the neural networks were not tuned but chosen according to the phenomenon of the over-parameterization generalization ability [52]. The hyperparameters were kept the same for all training, to avoid overfitting the hyperparameters. ReLU activation functions were chosen to preserve some linearity but also allow non-linear flexibility [53].

### Training and validation

Our two main centenarian clocks were trained in individuals aged 40 + . We also trained clocks in people aged in different groups, e.g., 90 + or 80 + , but found that this did not improve the performance. Therefore, we focused on presenting results from the analysis of 40 + and 100 + only.

For both EN and NN, we have a sample weight of 10 for the centenarian samples. After training to convergence, we perform random splitting 20-fold cross-validation for an unbiased validation.

### Clock software

The elastic net-based centenarian clock can be found in the Supplement. The neural network-based software can be downloaded from Github. https://github.com/victorychain/Centenarian-Clock.git

### Meta-analysis

We used the R function metafor for the fixed effect meta-analysis models weighted by inverse variance [54]. We combine the study results across the following cohorts: FHS, WHI BA23, and JHS.

### Diet, clinical biomarkers, and lifestyle factors

We performed a robust correlation analysis (biweight midcorrelation, bicor [27]) between the epigenetic age acceleration measures of three centenarian clocks and a total of 59 variables including 27 self-reported diet, 9 dietary biomarkers, 17 clinically relevant measurements, and 6 lifestyle factors including hand grip strength. The sample size for each variable is up to 6397 individuals across FHS, WHI BA23, and JHS cohorts. The 9 dietary biomarkers are only available in the WHI cohort. Blood biomarkers were measured from fasting plasma collected at baseline. Food groups and nutrients are inclusive, including all types and all preparation methods; e.g., folic acid includes synthetic and natural, and dairy includes cheese and all types of milk. The individual variables of WHI are explained in our previous study [26]. For each study cohort, we stratified the samples based on ethnic-gender category. The WHI samples were stratified by European, African, and Hispanic ancestry groups. Ancestry information was verified using ancestry informative SNP markers. We conducted robust correlation (bicor) analysis stratified by study cohort/ethnicity/sex and meta-analyzed the results with fixed effect models weighted by inverse variance. The fixed effect models yield a meta-estimate of bicor. As a caveat, the bicor analysis did not accommodate the intra-pedigree correlation in FHS.

### Universal chromatin state analysis

We used a recently published universal ChromHMM chromatin state annotation of the human genome [33]. The underlying hidden Markov model (HMM) was trained with over 1000 data sets of 32 chromatin marks in more than 100 human cell and tissue types. This model then produced a single chromatin state annotation per genomic position that is applicable across cell and tissue types, as opposed to producing an annotation that is specific to one cell or tissue type. A total of 100 distinct states were generated and categorized into 16 major groups according to the parameters of the model and external genome annotations [33].

We performed a one-sided hypergeometric analysis to study both the enrichment (odds ratios [OR] > 1) and depletion (OR < 1) patterns for our age-related markers based on the top 1000 CpGs with a positive correlation with age and the top 1000 CpGs with a negative correlation with age. Of the 33,495 CpGs used in our EWAS, 33,340 sites could be annotated to a unique chromatin state and were remained in our analysis. The background in our hypergeometric analysis was specified based on the 33,340 CpGs.

### Polycomb repressive complex

We defined indicator variables for PRC annotations based on the binding of at least two members of polycomb repressor complex 1 (PRC1 with subgroups RING1, RNF2, BMI1) or PRC2 (PRC2 with subgroups EED, SUZ12, and EZH2). Of the 33,340 CpGs, 4.25% sites were located near binding sites of PRC1, and 21.38% sites were located near binding sites of PRC2.

### DNA extraction and bisulfite conversion

Extraction of genomic DNA from blood was performed using the AllPrep DNA/RNA/protein kit (QIAGEN, Hilden, Germany). Sodium bisulphite conversion for Infinium HumanMethylation450 beadchip was performed using the EZ-DNA methylation-gold kit and the EZ-96 DNA methylation kit, respectively, and genome-wide DNA methylation was analyzed using the Infinium HumanMethylation450 beadchip (Illumina, San Diego, CA) following the manufacturer’s instructions. Arrays were scanned by HiScan (Illumina).

### Betterhumans study

James Clement engaged in an extensive, international study of centenarians, supercentenarians, and their offspring. The DNA samples were provided by Betterhumans Inc. a 501(c)(3), tax-exempt scientific research organization.

The object of the Betterhumans Supercentenarian Research Study is to compare genomic and molecular data from extremely long-lived individuals with “normal” shorter-lived individuals, especially those who died having known illnesses, such as cancer, cardiovascular diseases, Alzheimer’s, stroke, and diabetes,

https://www.supercentenarianstudy.com/

### Italian centenarian data

The individuals were recruited in three Italian centers (Bologna, Milan, and the University of Calabria at Rende). This data set (measured on the Illumina 450 K array) includes 192 subjects: 82 semi-supercentenarians (33 from Bologna, 29 from Milan, and 20 from Calabria), 63 offspring of semi-supercentenarians (22 from Bologna, 28 from Milan, and 13 from Calabria) and 47 control subjects whose parents were not centenarians (16 from Bologna, 17 from Milan and 14 from Calabria).

### CRELES cohorts

We used both data generated from the 450 K array and the EPIC array from participants of the “CRELES-Costa Rican study of longevity and healthy aging” from the Costa Rican Berkeley CRELES cohort. The 450 K array data were applied to whole blood and were collected from 95 individuals. The EPIC data were applied to whole blood samples collected from 508 individuals who are participants of the “CRELES-Costa Rican study of longevity and healthy aging” [55, 56].

### The Framingham Heart Study (FHS)

The FHS cohort [57] is a large-scale longitudinal study started in 1948, initially investigating the common factors of characteristics that contribute to cardiovascular disease (CVD). The study initially enrolled participants living in the town of Framingham, Massachusetts, who were free of overt symptoms of CVD, heart attack, or stroke at enrollment. In 1971, the study started the FHS offspring cohort to enroll a second generation of the original participants’ adult children and their spouses (*n* = 5124) to conduct similar examinations [58]. Participants from the FHS offspring cohort were eligible for our study if they attended both the seventh and eighth examination cycles and consented to having their molecular data used for the study. We used the 2544 participants from the group of health/medical/biomedical (IRB, MDS) consent with available DNA methylation array data.

Deaths among the FHS participants that occurred prior to January 1, 2013, were ascertained using multiple strategies, including routine contact with participants for health history updates, surveillance at the local hospital and in obituaries of the local newspaper, and queries to the national death index. Death certificates, hospital and nursing home records prior to death, and autopsy reports were requested. When cause of death was undeterminable, the next of kin were interviewed. The date and cause of death were reviewed by an endpoint panel of 3 investigators.

All participants provided written informed consent at the time of each examination visit. The study protocol was approved by the Institutional Review Board at Boston University Medical Center (Boston, MA).

#### DNA methylation

Peripheral blood samples were collected at the 8th examination. Genomic DNA was extracted from the buffy coat using the Gentra Puregene DNA extraction kit (Qiagen) and bisulfite converted using the EZ DNA methylation kit (Zymo Research Corporation). DNA methylation quantification was conducted in two laboratory batches using the Illumina Infinium HumanMethylation450 array (Illumina). Methylation beta values were generated using the bioconductor minfi package with Noob background correction.

### Women's Health Initiative (WHI)

The WHI is a national study that enrolled postmenopausal women aged 50–79 years into the clinical trials (CT) or observational study (OS) cohorts between 1993 and 1998 [59, 60]. We included 2107 WHI participants with available phenotype and DNA methylation array data from “Broad Agency Award 23” (WHI BA23). WHI BA23 focuses on identifying miRNA and genomic biomarkers of coronary heart disease (CHD), integrating the biomarkers into diagnostic and prognostic predictors of CHD and other related phenotypes, and other objectives can be found in https://www.whi.org/researchers/data/WHIStudies/StudySites/BA23/Pages/home.aspx.

### Jackson Heart Study (JHS)

The JHS is a large, population-based observational study evaluating the etiology of cardiovascular, renal, and respiratory diseases among African Americans residing in the three counties (Hinds, Madison, and Rankin) that make up the Jackson, Mississippi, metropolitan area [61].

The age at enrollment for the unrelated cohort was 35–84 years; the family cohort included related individuals > 21 years old. Participants provided extensive medical and social history, had an array of physical and biochemical measurements and diagnostic procedures, and provided genomic DNA during a baseline examination (2000–2004) and two follow-up examinations (2005–2008 and 2009–2012). The annual follow-up interviews and cohort surveillance are ongoing. In our analysis, we used the visits at baseline from 1747 individuals as part of project JHS ancillary study ASN0104, available with both phenotype and DNA methylation array data.

#### DNA methylation quantification

In brief, bisulfite conversion using the Zymo EZ DNA methylation kit (Zymo Research, Irvine, CA, USA) as well as subsequent hybridization of the HumanMethylation450k beadchip (Illumina, San Diego, CA) and scanning (iScan, Illumina) was performed according to the manufacturers protocols by applying standard settings. DNA methylation levels (*β* values) were determined by calculating the ratio of intensities between methylated (signal A) and un-methylated (signal B) sites. Specifically, the *β* value was calculated from the intensity of the methylated (M corresponding to signal A) and un-methylated (U corresponding to signal B) sites, as the ratio of fluorescent signals *β* = Max(M,0)/[Max(M,0) + Max(U,0) + 100]. Thus, *β* values range from 0 (completely un-methylated) to 1 (completely methylated). Peripheral blood samples were collected at the baseline. Methylation beta values were generated using the bioconductor *minfi* package with Noob background correction [62].

## Supplementary Information

Below is the link to the electronic supplementary material.

Supplementary file1 (PDF 1176 KB)

Supplementary file2 (CSV 20 KB)

Supplementary file3 (XLSX 671 KB)

## Data Availability

The WHI data (BA23) are available through dbGAP (dbGaP Study Accession: phs001335.v1.p3). The FHS data are available at dbGaP under the accession numbers phs000342 and phs000724. JHS data is available on dbGaP at phs000286, as well as by manuscript proposal request at https://www.jacksonheartstudy.org/. Due to confidentiality concerns, there are limitations on data access to the centenarian data. Please contact Steve Horvath (shorvath@mednet.ucla.edu) regarding access to the methylation data.
