# Supplementary material for: Centenarian clocks: epigenetic clocks for validating claims of exceptional longevity
Source: GeroScience. 2023 Mar 25;45(3):1817–35. doi: 10.1007/s11357-023-00731-7 (PMC10400760; doi:10.1007/s11357-023-00731-7)
Supplement: Supplementary file 1 — Supplementary file1 (PDF 1176 KB) [file 11357_2023_731_MOESM1_ESM.pdf]

Supplementary Material

for the article entitled
"Centenarian clocks: epigenetic clocks for validating claims of exceptional longevity" by Eric Dec et al.

Supplementary Figures

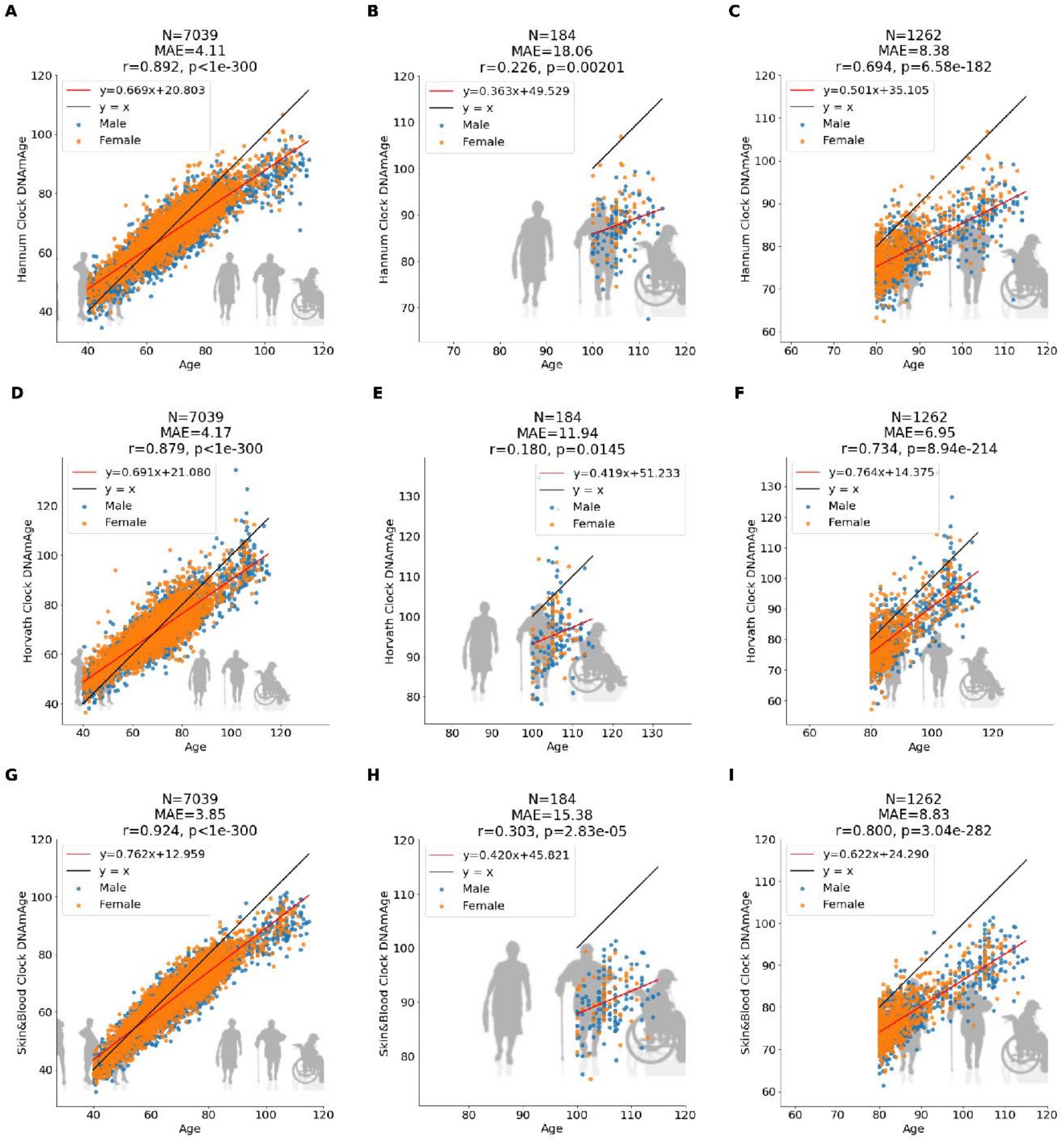

Supplementary Figure S1. Previously developed epigenetic clocks applied to the data set. This figure is analogous to our main Figure 1 but the rows present results for previously published epigenetic clocks. A,B,C) Blood based methylation clock from Hannum 2013. D,E,F)

Pan tissue clock from Horvath 2013. G,H,I) Skin & Blood Clock from (Horvath 2018). Each column corresponds to a different age range. DNA methylation data from age 40 to 115 (A,D,G), 100 to 115 (B,E,H), and 80 to 115 (C,F,I). Each panel reports the sample size (N), the median absolute error (MAE), Pearson correlation coefficient (r), the p-value (p), and each point is color coded by sex (blue=male).

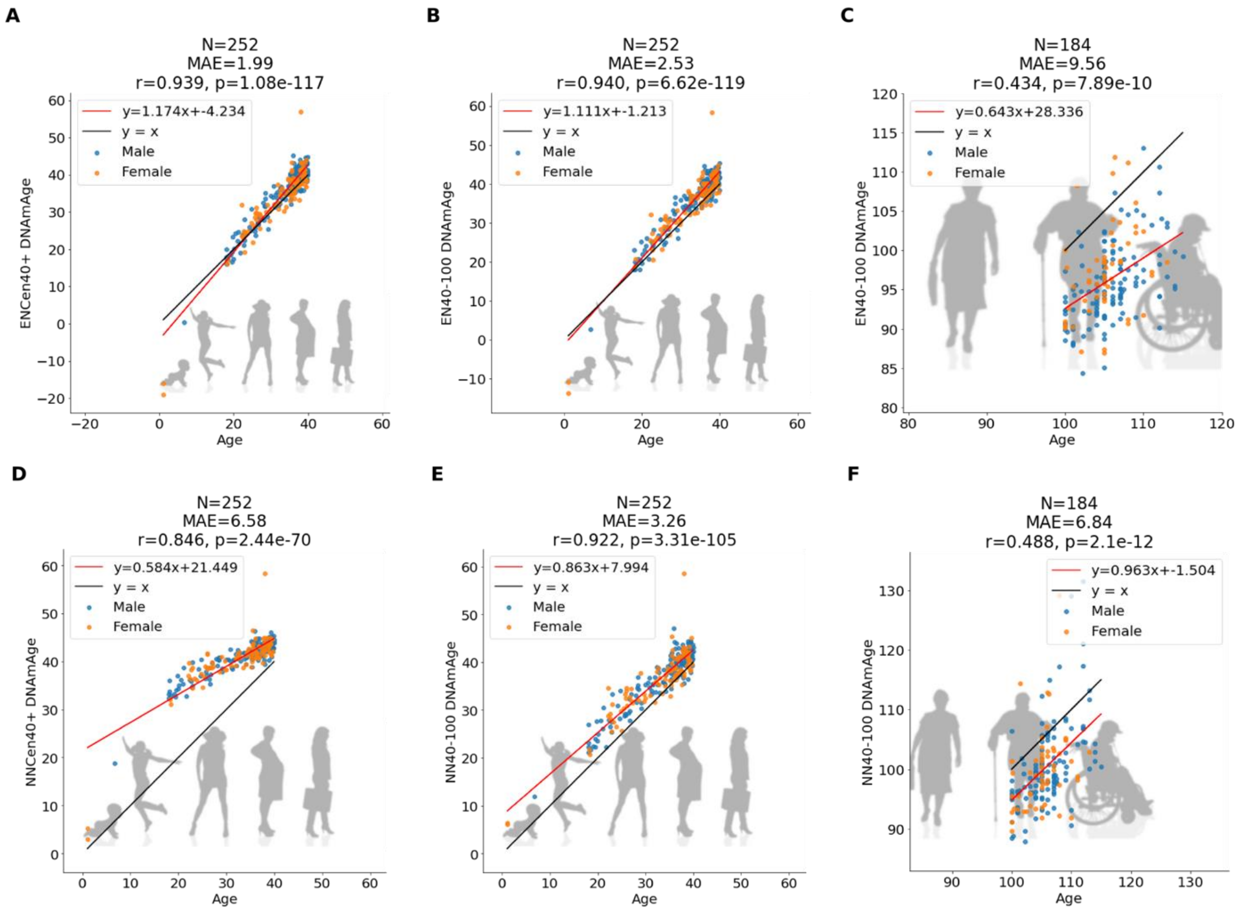

Supplementary Figure S2. Out-of-distribution analysis of clocks. To evaluate the generalizabilities/transportability of clocks to age ranges outside those employed in the the training set, we trained new clocks in age ranges specified in the names on the y-axis. For example, 40+ indicates that the clock was trained in individuals aged 40+. Similarly, 40-100 indicates that the clock was trained in individuals aged between 400 and 100. The x-axis reports the chronological age in the two test sets: 1) young age group defined as age<40 and 2) old age group defined by 100+. This analysis uses ElaticNet (top row A,B,C) and Neural Network (bottom row D,E,F) trained on certain age ranges, 40 to 115 (A,D), 40 to 100 (B,C,D,F), to make predictions on age ranges that are not in the training age range, 0 to 40 (A,B,D,E), 100 to 115 (C,F)

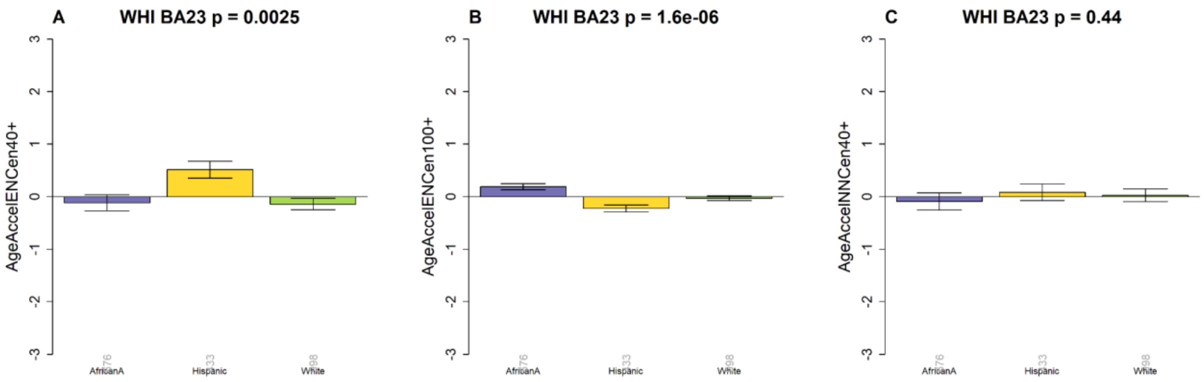

Supplementary Figure S3 Association between age acceleration measures and ethnicity. The figure presents bar plots for the associations between ethnic/racial group and age acceleration measures of A) ENCen40+, B) ENCen100+, and C) NNCen40, respectively, using WHI BA23 cohort. Each title presents the P value based on non parametric Kruskal -Wallis test. The x-axis lists three racial groups: African American, Hispanic and White. The number under each bar presents number of individuals at each racial group.

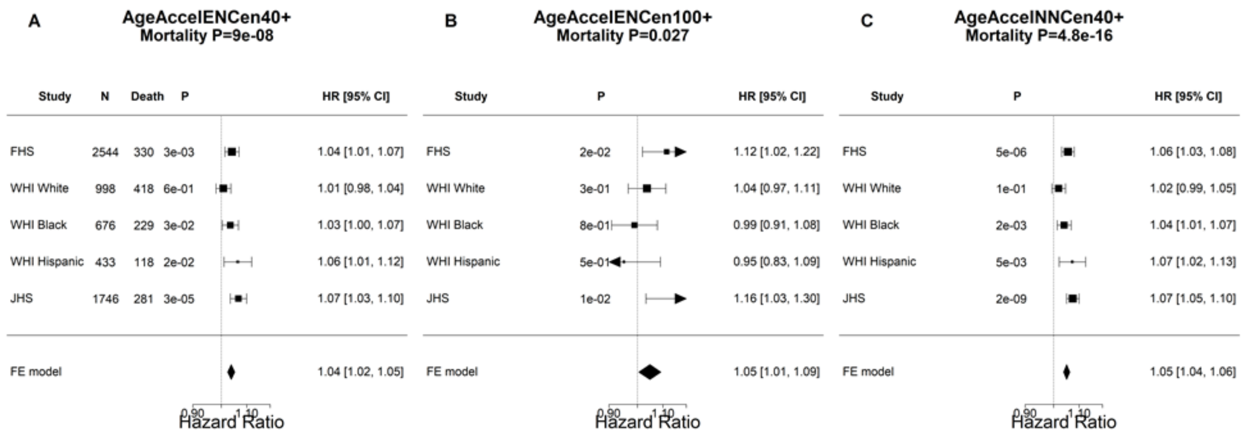

Supplementary Figure S4. Centenarian clocks predict all-cause mortality. The figures present forest plots for predicting time-to-death due to all-cause mortality. Fixed effect meta analysis was performed to combine mortality analysis across five results from three study cohorts stratified by racial group: Framingham Heart Study (FHS), Women's Health Initiatives (WHI) BA23, and Jackson Heart Study (JHS). We regressed each centenarian clock on chronological age and used the residuals to form an age acceleration measure (AgeAccel) in units of years. Each panel reports a meta analysis forest plot for combining hazard ratios predicting time-to-death based on AgeAccel of A) ENCen40+, B)ENCen100+ and C)NNCen40+ clock, respectively. Each row reports a hazard ratio (for time-to-death) and a 95% confidence interval resulting from a Cox regression model in each of 5 strata. The sub-title of each panel reports the meta analysis P-value. Each hazard ratio (HR) corresponds to a one-year increase in AgeAccel.

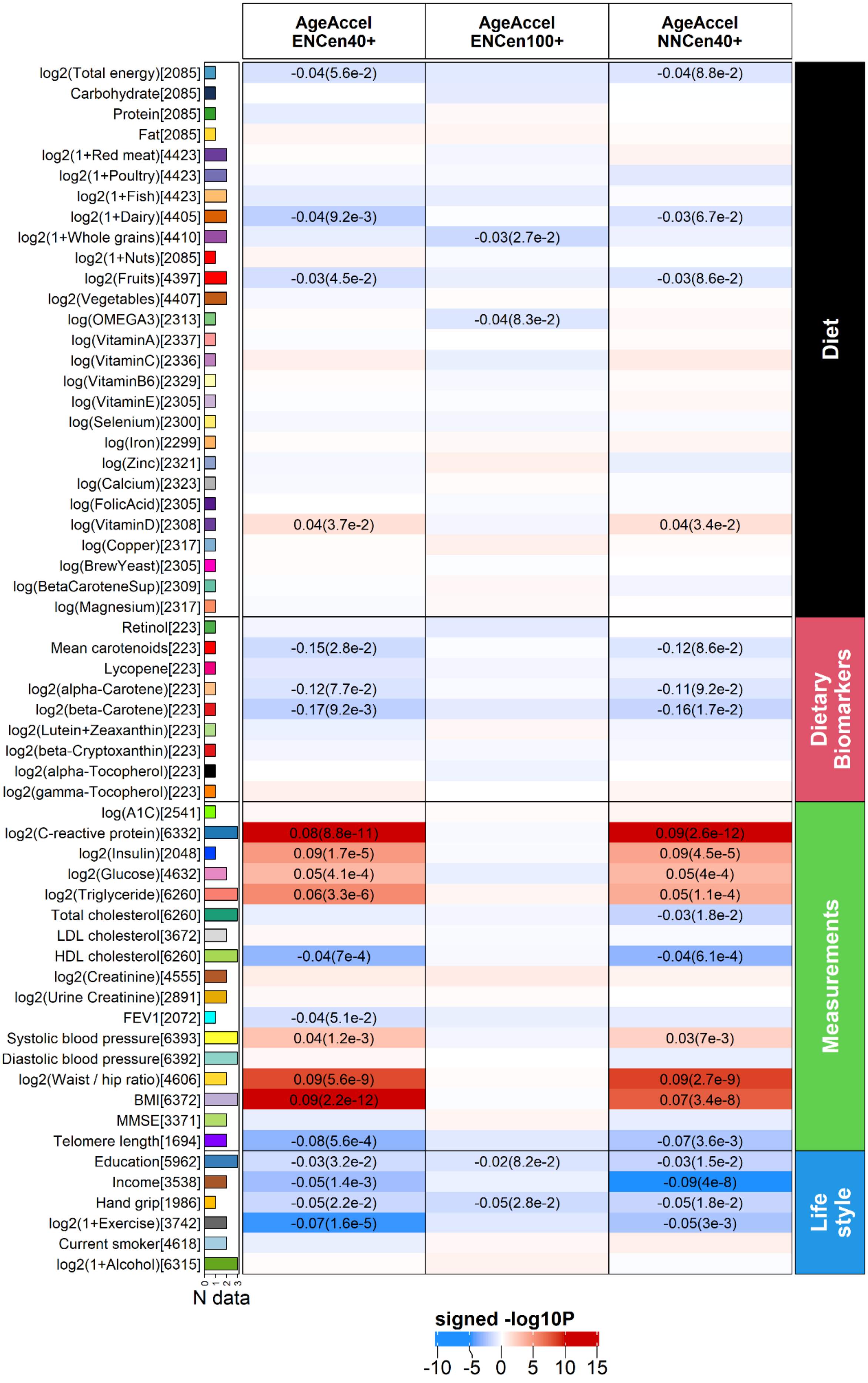
AgeAccel ENCen40+
AgeAccel ENCen100+
AgeAccel NNCen40+
log2(Total energy)[2085]
Carbohydrate[2085]
Protein[2085]
Fat[2085]
log2(1+Red meat)[4423]
log2(1+Poultry)[4423]
log2(1+Fish)[4423]
log2(1+Dairy)[4405]
log2(1+Whole grains)[4410]
log2(1+Nuts)[2085]
log2(Fruits)[4397]
log2(Vegetables)[4407]
log(OMEGA3)[2313]
log(VitaminA)[2337]
log(VitaminC)[2336]
log(VitaminB6)[2329]
log(VitaminE)[2305]
log(Selenium)[2300]
log(Iron)[2299]
log(Zinc)[2321]
log(Calcium)[2323]
log(FolicAcid)[2305]
log(VitaminD)[2308]
log(Copper)[2317]
log(BrewYeast)[2305]
log(BetaCaroteneSup)[2309]
log(Magnesium)[2317]
Retinol[223]
Mean carotenoids[223]
Lycopene[223]
log2(alpha-Carotene)[223]
log2(beta-Carotene)[223]
log2(Lutein+Zeaxanthin)[223]
log2(beta-Cryptoxanthin)[223]
log2(alpha-Tocopherol)[223]
log2(gamma-Tocopherol)[223]
log(A1C)[2541]
log2(C-reactive protein)[6332]
log2(Insulin)[2048]
log2(Glucose)[4632]
log2(Triglyceride)[6260]
Total cholesterol[6260]
LDL cholesterol[3672]
HDL cholesterol[6260]
log2(Creatinine)[4555]
log2(Urine Creatinine)[2891]
FEV1[2072]
Systolic blood pressure[6393]
Diastolic blood pressure[6392]
log2(Waist / hip ratio)[4606]
BMI[6372]
MMSE[3371]
Telomere length[1694]
Education[5962]
Income[3538]
Hand grip[1986]
log2(1+Exercise)[3742]
Current smoker[4618]
log2(1+Alcohol)[6315]
-0.04(5.6e-2)
-0.04(8.8e-2)
-0.04(9.2e-3)
-0.03(6.7e-2)
-0.03(2.7e-2)
-0.03(4.5e-2)
-0.03(8.6e-2)
-0.04(8.3e-2)
0.04(3.7e-2)
0.04(3.4e-2)
-0.15(2.8e-2)
-0.12(8.6e-2)
-0.12(7.7e-2)
-0.11(9.2e-2)
-0.17(9.2e-3)
-0.16(1.7e-2)
0.08(8.8e-11)
0.09(2.6e-12)
0.09(1.7e-5)
0.09(4.5e-5)
0.05(4.1e-4)
0.05(4e-4)
0.06(3.3e-6)
0.05(1.1e-4)
-0.03(1.8e-2)
-0.04(7e-4)
-0.04(6.1e-4)
-0.04(5.1e-2)
0.04(1.2e-3)
0.03(7e-3)
0.09(5.6e-9)
0.09(2.7e-9)
0.09(2.2e-12)
0.07(3.4e-8)
-0.08(5.6e-4)
-0.07(3.6e-3)
-0.03(3.2e-2)
-0.02(8.2e-2)
-0.03(1.5e-2)
-0.05(1.4e-3)
-0.09(4e-8)
-0.05(2.2e-2)
-0.05(2.8e-2)
-0.05(1.8e-2)
-0.07(1.6e-5)
-0.05(3e-3)
Diet
Dietary Biomarkers
Measurements
Life style
0 1 2 3
N data
signed -log10P
-10 -5 0 5 10 15

Supplementary Figure S5. Meta cross-sectional correlations between centenarian clocks and a host of measurements. Robust correlation coefficients (1) between 1) age acceleration measure of centenarian clocks, and 2) 59 variables including 27 self-reported diet, 9 dietary biomarkers, 17 clinically relevant measurements related to vital signs, metabolic traits, inflammatory markers, cognitive function, lung function, central adiposity and leukocyte telomere length (LTL), and 6 lifestyle factors such as education and hand grip strength. The y-axis lists variables in the format of name (sample size), followed by a bar plot denoting number of studies. Inverse variance weighting fixed effect models were applied to combine results across studies. Variables are arranged by category displayed on the right annotation. The x-axis lists AgeAccelENCen100+, AgeAccelENCen40+ andAgeAccelNNCen40+. Each cell presents meta bicor estimates and P-value, provided P<0.1. The color gradient is based on -log10 P-values times sign of bicor (1).

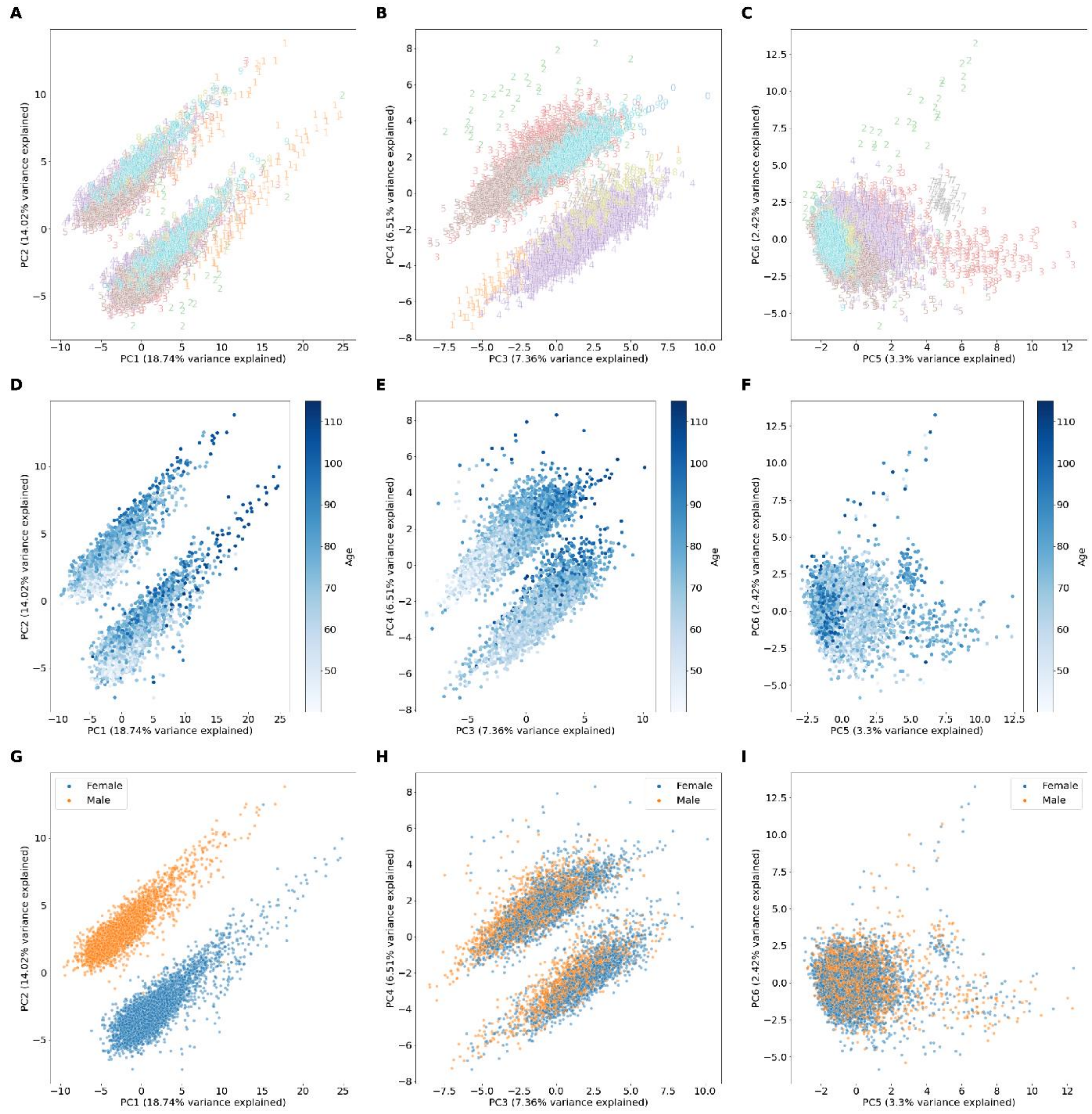

Supplementary Figure S6. Principal component analysis of the data set. Each point correspond to a DNA sample in the training set. The panels depict different combinations of principal components. (A,D,G) PC1 (x-axis) versus PC2 (y-axis). (B,E,H) PC3 versus PC4. C,F,I) PC5 versus PC6. The points are colored by (A,B,C) the underlying data set, (D,E,F) chronological age, (G,H,I), sex. A,B,C) Samples are labelled by data set. Our methylation data came from different sources (Table 1, Supplementary Table 1).

Supplementary Table

Study	Race[1]	Samples	Female	Death	Age	Follow-up	Array[2]
FHS	White	2544	54%	13%	66.3±8.62 [60,73]	7.8±1.70 [7.4,8.9]	450k
WHI BA23	White	998	100%	42%	68.3±6.26 [65,72.77]	16.8±4.58 [15,19.9]	450k
	AfricanA	676	100%	34%	63±6.61 [57.9,67.7]	16.7±5.04 [15.8,19.9]	
	Hispanic	433	100%	27%	62.2±6.87 [56.5,67.5]	17.5±4.02 [17.7,19.9]	
JHS	AfricanA	1746	63%	16%	56.2±12.32 [46.5,65.35]	11.7±2.55 [11.2,13.1]	EPIC

[1]AfricanA denotes African American.
[2]All DNA methylation data were profiled in Illumina arrays.

Supplementary Table 1: Study characters of human cohorts. The table summarizes the characteristics of 6,397 individuals from Framingham Heart Study Offspring Cohort (FHS), Women's Health Initiatives (WHI) BA23 and Jackson Heart Study (JHS). The study cohorts were used to evaluate our centenarian clocks in downstream analysis. Age and follow-up time (from methylation profile to last visit/death in units of years) are presented in the format of mean ±SD [25th, 75th].

References

1. Langfelder P, Horvath S. WGCNA: an R package for weighted correlation network analysis. BMC Bioinformatics. 2008;9(1):559.
